# Cognitive linguistics approach to the representation of knowledge contents in college English textbooks

**DOI:** 10.3389/fpsyg.2026.1749701

**Published:** 2026-02-16

**Authors:** Li Zhang, Zaishu Mo

**Affiliations:** School of Foreign Languages, Hunan University, Changsha, Hunan, China

**Keywords:** analysis model, cognitive linguistics, college English textbooks, conceptual metaphor theory, textbook analysis

## Abstract

This paper proposes a metaphor-based textbook analysis model from a macroscopic perspective and abstracts it with the “Source–Path–Goal (SPG)” image schema. Seeking new insights from cognitive linguistic theory, as well as conceptual metaphor theory (CMT), this research probes into the source, path, and goal of the metaphorical representation of knowledge contents in a series of college English textbooks in China. With the construction of a self-built textbook corpus and the aid of an online corpus annotation tool, qualitative content analysis was carried out to uncover the metaphorical representations in the textbook texts and tasks. Findings show that first, the conceptual metaphors in the texts and the metaphorical analogical reasoning in tasks are considered in the analysis model as “Source.” Second, structured design including the “hourglass” pattern in the unit design is the “Path” of knowledge representation in the textbooks. Third, the functions of these metaphorical representations are discussed as the “goal” of the knowledge representations in the textbooks, which is the realization of competence development as the textbooks’ teaching objectives. Since conceptual metaphor theory puts forward a universality of metaphors, the case study is not only typical in the Chinese context but also potentially informative beyond it.

## 1 Introduction

Although research on college English textbooks flourished in the 1990s, it has been largely overshadowed by scholarly enthusiasm for other aspects of English Language Teaching (ELT) in China. As a result, significant gaps persist in this area—particularly in light of the evolving national demands for talent amid current global circumstances. The long-standing influence of the grammar-translation method in China’s college English education has led to the design of traditional textbooks guided by structuralist principles. Such an approach, as noted by Richards and Rogers (2001, p. 5), “codifies the foreign language into frozen rules of morphology and syntax to be explained and eventually memorized.” The overemphasis on linguistic forms and grammatical accuracy reduces the fun of language learning to rote memorization and mechanical drills, ultimately leading to low instructional efficiency, limited development of critical thinking, and inadequate communicative competence.

The idea that “we do not have direct access to the brain” while it’s working makes the psychological process of language learning even more attractive to psychologists and linguists. The psychological process of language and its cognitive development has been “one of the most intriguing subjects in science” (Banaruee et al., 2023, p. 1, 6). As a direct medium for learning activity, textbooks will theoretically be more effective if developed and improved with human basic cognitive abilities as the starting point, aligned more closely with learners’ cognitive patterns, and taking cognitive rules as the core medium for language learning. Cognitive linguistics and the related theories and principles give provide insights into language teaching and learning, considering language as part of human cognitive development and focusing on the underlying cognitive mechanisms of language (Pütz et al., 2001; Holme, 2009; Boucheix et al., 2020).

However, on the whole, “there is still much blank space for the application of the core concepts of cognitive linguistics in foreign language pedagogy, such as in teacher research, textbook research, assessment research, etc. (Xu, 2023, p. 83).”

Starting from these initiatives, this paper constructed a textbook analysis model employing cognitive linguistic principles. The purpose is to integrate textbook research and development with cognitive linguistic principles and theories to be “aware of the pedagogical implications of current theory and research in linguistics and language learning” and to achieve “a broader educational concern” (Sheldon, 1988, p. 239).

## 2 Literature review

The application of linguistic principles and theories to textbook research witnesses textbook evaluation against the national teaching syllabus in the Chinese context. The educational concern lies with the college English structural curriculum developed under the influence of Structuralist linguistics, which set its teaching objectives on language structures, grammar rules, and the training of reading ability, and was dominant in ELT in China in the 1980s (Shu, 2024; Li, 2021). Scholarly attention paid to the psychological process and cognitive concerns of language acquisition (Banaruee et al., 2023) gives initiative to this study.

### 2.1 Cognitive–linguistic approaches to textbook research

A recent forum at the 8th Workshop on Cognitive Linguistics and Second Language Acquisition (2024) emphasized the integration of cognitive linguistics into textbook design. Researchers from Shanghai International Studies University demonstrated how key theories such as construction grammar and schema theory are being actively implemented in the development of language textbooks for primary, junior, and senior high school students (Tian, 2024; Liu, 2024; Ren, 2024). Such attempts in textbook compilation provide textbook evaluation from a cognitive linguistic perspective, though the latter is still in an infant stage. It presents theoretical as well as applicable research gaps for this study to consider.

Drawing on Littlejohn’s (2011, p. 183) framework for analyzing language teaching materials, which builds upon the approach established by Richards and Rodgers (2001), this paper focuses particularly on the “design” level. This dimension captures the underlying rationale of textbooks, including their aims, principles of knowledge content selection and sequencing, subject matter, and types of learning activities (Deng and Wang, 2023). By applying these criteria to the content analysis of college English textbooks, this study seeks to uncover the representation and realization of language knowledge in the textbooks. Examining how tasks and texts are represented, and in what ways the stated aims align with actual material construction, can reveal how theoretical principles are operationalized in practice, thus illuminating the realization of the compilation philosophy and the instructional implementation.

### 2.2 Conceptual metaphor theory and metaphor-based textbook analysis

Metaphor studies in China gained significant momentum in the 1990s, largely due to the introduction and popularization of Lakoff and Johnson’s conceptual metaphor theory (CMT). CMT has made several foundational arguments that have profoundly influenced the research field:

The first is the ubiquity of metaphor: “Metaphor is pervasive in everyday life, not just in language but in thought and action (Lakoff and Johnson, 1980/2003, p. 4).”

The second is the systematic nature of metaphor: Metaphorical concepts are inherently systematic in nature, thus enabling individuals to comprehend one domain of experience in terms of another. As the metaphorical concept is systematic in nature, the language employed to articulate this aspect of the concept is also systematic (ibid: 8). The systematicity in question has been demonstrated to facilitate coherent and structured thinking, and the inherent comprehension mechanism of metaphors also demonstrates its efficiency in language acquisition (Banaruee et al., 2019; Banaruee et al., 2023).

The third is the classification of conceptual metaphors: CMT has categorized conceptual metaphors into three types. Banaruee et al. (2019) then developed two types of metaphors with two comprehension mechanisms. Metaphor comprehension has utilized embodied cognition and the sensory motion system ever since the early period of language acquisition.

The inquiry into textbook analysis and evaluation of metaphor usage encompasses two major tenets.

First, the use of conceptual metaphor and grammatical metaphor in the textbooks was analyzed to reveal their linguistic manifestations, distribution patterns, and pedagogical implications based on comprehensive analysis, corpus-based methodology, and systematic investigation (Gao and Wang, 2021; Chen and Wu, 2015; Sznajder, 2010). Evaluation of effectiveness was carried out on business English textbooks against the teaching syllabus (Sun and Wang, 2013). Second, a metaphor questionnaire survey was conducted to learn about the users’ attitudes toward the textbooks in use (Kesen, 2010; Rathert and Cabaroglu, 2021; Han, 2014; Liu and Jiao, 2021). Comprehensive research for a closer examination of the use of metaphor in textbooks is lacking in this field.

### 2.3 Metaphor identification methods

Empirical studies on metaphor identification provide essential data for analyzing metaphors in discourse, enabling researchers to explore how metaphors function in various contexts. The three principal experimental methods in the field of metaphor research are the identification of metaphors, their comprehension, and the production of metaphors. Metaphor identification is widely regarded as the cornerstone of this tenet, as it provides the foundational data necessary for understanding how metaphors are processed and utilized in both language and thought.

Researchers have progressively refined how metaphors are identified. Zhong and Chen (2013) systematized the field by outlining five main approaches: form, logic, reference, multiple dimensions, and conceptual structure. Following this, Liu and Wang (2017) focused on the practical techniques, classifying the identification process into three types based on the level of human involvement: manual, semi-automated, and fully automated.

They also elaborated on specific identification criteria, including metaphor discourse markers (Goatly, 1997), semantic incongruity (Kittay, 1984), comparative mode (Liu and Cao, 2017; Steen et al., 2010; Pragglejaz Group, 2007), machine learning techniques (Ge et al., 2023; Zhang, 2019; Gedigian et al., 2006), and corpus-based tools (Alharbi, 2025). Together, these criteria and methods represent the evolving landscape of metaphor identification research, highlighting the integration of traditional linguistic analysis with advanced computational approaches. Together, these studies emphasize the methodological diversity and interdisciplinary nature of metaphor research, providing valuable tools for theoretical exploration and practical discourse analysis.

However, manual identification has faced significant criticism. Manual identification relies heavily on researchers’ metalinguistic intuition and subjective judgment, which may not accurately reflect the cognitive processes underlying metaphor use. Consequently, its validity and reliability have been called into question (Kövecses, 2011). In response to these limitations, a cross-disciplinary approach involving natural language processing (NLP)-driven metaphor identification has emerged, offering greater specificity and objectivity. However, despite its advantages, NLP techniques present substantial challenges for language researchers, particularly in terms of technical complexity and the need for interdisciplinary expertise. Furthermore, these methods often lack the flexibility required to adapt to diverse linguistic and cultural contexts, which limits their applicability in certain scenarios (Cao and Liu, 2022, p. 10). Therefore, although NLP-driven approaches are a significant advancement in metaphor identification, they have their own limitations, which highlight the need for continued methodological innovation and interdisciplinary collaboration.

The semi-autonomous approach to metaphor identification is characterized by its development from a theoretical framework into a robust and widely applicable methodology. The approach itself was systematically categorized as part of a larger evolution in identification techniques (Liu and Wang, 2017), emerging as a hybrid solution that combines the efficiency of computational corpus tools with the nuanced judgment of manual analysis. A key technical advancement in this method is the use of specialized software like Wmatrix. Its sophisticated semantic annotation capability specifically addresses a critical limitation of purely manual checking by reliably identifying vocabulary associated with the source and target domains of conceptual metaphors (Chen, 2022).

This advancement has been validated through successful empirical application. The semi-autonomous method has proven its versatility and reliability by being effectively deployed to analyze metaphorical patterns across a diverse range of genres, including news media, economic and political discourse, and educational materials (Xin and Shan, 2018; Sun, 2012; Zhang and Miao, 2012). Consequently, it has established itself as a scientifically sound approach for uncovering the cognitive and linguistic dimensions of metaphorical language.

Inspired by these advancements, this study takes a revised semi-automated approach to analyze textbooks. The aim is to contribute to a deeper understanding of how metaphors function in pedagogical contexts and provide insights to improve the design and evaluation of language teaching resources.

### 2.4 Motion-based conceptualization of “textbooks”

Cognitive linguistics takes conceptual metaphor as a common way of human thinking, which people use as cognitive mediation to conceptualize unfamiliar constructs. Metaphor manifests high efficiency in promoting comprehension, developing creative thinking, and applying theories to the study of language structure as well as linguistic discourse (Gao and Zhu, 2021, Hart and Lukeš, 2007).

In metaphorical representation, motion domains are applied to describe non-motion events (Khatin-Zadeh et al., 2017; Banaruee et al., 2019), for “motion events are the ubiquitous feature of our daily lives. Almost every corner of our lives includes some kind of motion activity (Banaruee et al., 2019, p. 3).” A typical example is the metaphorical description of the emotional state of anger in “He went up and came down” (Khatin-Zadeh et al., 2017, p. 120). Cross-linguistic evidence is given by Eskandari and Khatin-Zadeh (2021) to demonstrate the routine use of metaphor in describing abstract emotions by human beings. Theoretical applications have been made by conceptualizing “time” with spatial movement (Banaruee et al., 2019) and framing a three-dimensional geometric representation of mathematical concepts (Khatin-Zadeh et al., 2017). Gentner and Gentner (1983) also contended that the mental process of electricity is akin to flowing water.

Motion events are effective in describing abstract concepts, and their cognitive embodied philosophy lies in early language acquisition through the sensorimotor system (Banaruee et al., 2019). Motion events are particularly suitable for metaphorically describing domains characterized by dynamic changes over time.

In the model where non-motion concepts are represented in terms of motion events, an abstract concept can be grasped through a concrete one by functioning as a perceptual intermediary, enabling comprehension via sensory experience (Banaruee et al., 2019, p. 2). This mechanism of interpreting the abstract through the concrete constitutes a form of representational transformation (Khatin-Zadeh et al., 2017). The motion event that enables such transformation should include several essential components: an object (the figure) moves from a starting point (the source) to an endpoint (the goal), following a specific trajectory (the path). Additional elements integral to any motion event include the manner of motion and the reference frame, or ground, against which the movement is described (Banaruee et al., 2019, p. 3).

Following this metaphorical model and enlightened by embodied cognitive philosophy, this study creatively treats the non-motion construct of textbooks as motion events, rigorously expanding the application of existing “path-centered” analyses in ELT textbooks from an inner textbook ontological viewpoint focusing on language features (Banaruee et al., 2023) to an outer, larger, and macroscopic perspective of textbook compilation and structural designs targeting structural relations and cognitive processes. The concept itself, its knowledge contents, and its design pattern boast the same components with structural similarities to “motion events” in the above model, which is depicted in Table 1.

**Table 1:** Components of motion events for the metaphorical representation of “textbook.”

Motion events: journey (source domain)					
Figure	Source	Manner	Goal	Ground	Path
Agent	Starting point	Transportation	Destination	Road, rail, or water	Route
Non-motion events: textbook (target domain)					
Figure	Source	Manner	Goal	Ground	Path
Compilers	Texts & tasks	A cross-unit design pattern	Teaching objectives	Compiling philosophy	Metaphorical representation

Structural similarities and component mapping from the abstract domain of textbooks to the concrete domain of motion events (such as journeys) provide the same embodied experience as Gibbs (2005)‘s “SPG” image schema. Consequently, the following study constructs the “SPG” schema to analyze a series of college English textbooks in the Chinese ELT context.

### 2.5 Knowledge contents and metaphorical representation of knowledge contents

Textbook writing is a practice-oriented endeavor that must be guided by a coherent compilation philosophy, informed by contemporary developments in linguistic theory and the essence of language learning. Such a philosophy, together with a well-structured compilation procedure, forms the foundation for achieving pedagogical objectives. As Richards and Rodgers (2001) suggested, language theory shapes teaching methodology, which in turn informs syllabus design and textbook content.

#### 2.5.1 Knowledge contents in textbooks

Research on knowledge contents in textbooks can be traced to the concept of “structured knowledge” introduced in the English Curriculum Standard for Ordinary Senior High School (2017 Edition, 2020 Revision) [Ministry of Education (MOE), 2020, pp. 69–73]. Structured knowledge in English is defined as an integrative representation of language, thinking, and culture, serving to systematically organize otherwise fragmented subject matter. Unlike traditional textbook designs—common in subjects such as mathematics—where knowledge is presented as isolated points, the revised curriculum standards advocate a shift toward a “grand-concept” paradigm. According to Zhu (1996), knowledge representation encompasses both organizational and applicable structures. This shift aligns with the principles of “holistic teaching” and “structured design” (Wang et al., 2022; Cho and Park, 2014), which will be elaborated in the following section. Furthermore, conceptual metaphor theory identifies structural metaphor as a primary category of metaphorical thought. To understand how metaphors of knowledge are represented, we can analyze their structural domain mappings in language, where they are pervasively present.

#### 2.5.2 Metaphorical representation of knowledge contents

As a core element of teaching materials, the “knowledge content” in textbooks, which is the selection of essential knowledge and principles, is predominantly shaped by the nature of the course (Deng and Wang, 2023). Recent research on the knowledge representation of textbooks is abundant in cultural contents (Zhang et al., 2024, Liu et al., 2022; Lu et al., 2022). Integrating educational guidelines of English teaching curriculum, this study puts forward the trinity of knowledge contents in college English textbooks, which includes language, critical thinking, and culture (You et al., 2019).

Within cognitive psychology and based on conceptual metaphor theory (Li, 2018), studies on the metaphorical representation of time reveal how abstract concepts are grounded in concrete experience as the motion events are metaphorically used for the description of non-motion constructs. We extend this principle to the concept of knowledge. Its representation in textbooks is not neutral but should be an instrumental pedagogical translation, deliberately structured to foster competency development and enable a positive transfer from pure subject matter to teachable knowledge content. Building on conceptual metaphor theory (CMT), this study theoretically posits a critical link between the metaphorical representation of knowledge content and the development of linguistic and cognitive competencies.

Research on knowledge representation in textbooks can be traced to the concept of “structured knowledge” introduced in the English Curriculum Standard for Ordinary Senior High School (2017 Edition, 2020 Revision) [Ministry of Education (MOE), 2020, pp. 69–73]. Structured knowledge in English is defined as an integrative representation of language, thinking, and culture, serving to systematically organize otherwise fragmented subject matter. Unlike traditional textbook designs—common in subjects such as mathematics—where knowledge is presented as isolated points, the revised curriculum standards advocate a shift toward a “grand-concept” paradigm. According to Zhu (1996), knowledge representation encompasses both organizational and applicable structures. This shift aligns with the principles of “holistic teaching” and “structured design” (Wang et al., 2022), the latter of which is elaborated in the following section. Furthermore, conceptual metaphor theory identifies structural metaphor as a primary category of metaphorical thought. To understand how metaphors of knowledge are represented, we can analyze their structural domain mappings in language, where they are pervasively present.

## 3 Research hypothesis and research questions

Conceptual metaphor theory (Lakoff and Johnson, 1980/2003, p. ix) holds the view that “metaphor is pervasive in everyday language and thought”, Lakoff et al. (1991, p. 52–53) defined that “any purposeful action is a journey, and “journey defines a path”. Gibbs (2005) structured it as a “Source–Path–Goal schema”, integrated with the cognitive model to represent non-motion constructs in terms of motion events (Eskandari and Khatin-Zadeh, 2021; Khatin-Zadeh et al., 2017; Gentner and Gentner, 1983).

A critical link between the metaphorical representation of knowledge content and the development of linguistic and cognitive competencies is posited in this study, and they are structurally mapped to this image schema, as mapping components shown in Table 1.

Starting from these initiatives, the present study extends the conventional research scope of CMT to textbook analysis and probes into the function of conceptual metaphor at both the linguistic level and cognitive realm. This study is based on the research hypothesis that regards metaphorical representation as the “manner” component in the “SPG” schema to carry out a comprehensive analysis and evaluation of the targeted textbooks. The metaphorical representation of knowledge contents in the texts and tasks is the “source” of the “SPG” schema, the teaching objectives are the “goal” of the schema, while how textbooks effectively achieve this goal and the design pattern to realize such metaphorical representation, in accordance with the textbooks’ compilation philosophy, are the “path” of the schema. Based on this hypothesis, four research questions are formulated:

1. What is the metaphorical representation of knowledge content in the college English textbooks?
2. How is the knowledge contents metaphorically represented in the college English textbooks?
3. Why is the knowledge content metaphorically represented in the college English textbooks?

## 4 Materials and methods

In order to properly investigate the metaphorical representation of knowledge contents in the textbooks, this study mainly employs qualitative analysis based on deep reading of textbook materials and the method of content analysis facilitated by corpus tools.

### 4.1 Research materials

*The New Target College English Integrated Coursebooks (2nd Edition)* (hereafter referred to as “the Integrated Coursebooks”) have been selected as the case study in this research for three main reasons. First, as a series developed in accordance with the latest College English Teaching Guidelines, the second edition of *the Integrated Coursebooks* was revised and published following the official release of the 2020 Guidelines. It is distinctive in knowledge representation to exhibit a national necessity. Second, the textbooks claim to be informed by cognitive linguistic theories (Liu et al., 2016), making them a typical example of textbooks developed under the guidance of contemporary cognitive linguistic frameworks. The analysis will reveal their cognitive linguistic basis of compilation and serve as a potential reference for textbook development in other ELT contexts. Third, the compilation team consists primarily of faculty members from the authors’ own institution, a top university in China. It is beneficial to carry out organized research work and facilitate follow-up research and communication with the compilation team.

*The New Target College English Integrated Course* series comprises four volumes and is compiled by one chief editor and four co-writers. The topics covered include university life, social activities, diverse cultural contexts, as well as science and technology. The total corpus of the four volumes amounts to 61,434 words. There is a generalized unit structure and components of the textbooks:

Unit Title: represented with dual modes of both words and images;
Introduction: composed of brief theme introductions and theme-related Chinese vs. English quotations;
Warm-up activities: taking the form of audio presentations and included in the research materials as one of the tasks.
Reading articles: Text A and Text B discuss the unit theme, serving as the main resources of research materials and the content of the textbook corpus. They are included in the research as “Texts.”
Word lists: including new words, phrases, expressions, and proper names.
Reading Comprehension: questions and exercises about the two texts, taken as one of the “Tasks.”
Language In Use: lexical and grammatical exercises taking the form of blank-filling, matching, true–false decisions, and translation. They are considered the main content of “Tasks.”
Writing: including 3–4 exercises on writing techniques, ranging from paragraph writing to essay writing, which is included in the “Tasks.”
Project: a comprehensive activity encompassing group work, writing, presentations, and the like, targeted at the unit theme. It is included in the analysis of “Tasks.”

The above unit structure is a unified layout of a single unit in *the Integrated Coursebooks*. The discussion of texts refers to the two reading articles as Text A and Text B, and the analysis of tasks includes warm-up activities, reading comprehension, language in use, writing, and the project.

### 4.2 Research methods

The qualitative analysis in this study draws on a corpus-based approach. To address the first research question, selected college English textbooks were compiled into a specialized corpus, and linguistic metaphors were identified using Wmatrix, an online corpus tool.

A semi-automated metaphor annotation framework was adopted to mitigate the limitations of purely manual or fully automatic methods, which often yield subjective or imprecise results. Wmatrix (Rayson, 2011) enables part-of-speech tagging and semantic tagging, facilitating the identification of key semantic domains through comparison with a reference corpus.

Statistical significance was determined using a log-likelihood threshold of 6.63 (*p* < 0.001), corresponding to a 99% confidence level, with the BNC Sampler Written as the reference corpus. A useful tool of Wmatrix is USAS (UCREL Semantic Annotation System). The reference corpus is BNC Sampler Written, which is a subset of 968,267 words taken from the BNC Sampler and is mainly subjected to semantic annotation in this study using the USAS annotation system.

To further identify conceptual metaphors, manual annotation following MIPVU (Steen et al., 2010) was performed by two independent raters. The raters are student researchers on metaphor identification who were trained academically and technically on this topic and selected it as the topic of their graduation paper at a top-notch university in Hunan. Pre-training on metaphor identification was conducted in two sessions with their supervisor and professors. An English native speaker was invited as the final arbiter of disputes. Twenty percent of the manually identified metaphor-loaded linguistic expressions were randomly chosen for inter-rater reliability. Measured by Cohen’s kappa, the inter-rater reliability reached a Kappa value of 0.6879, indicating substantial agreement.

### 4.3 Research procedure

Data processing was conducted for one and a half months following the steps below:

1. Electronic materials of the reading materials were collected from the original digital materials from the compilers’ team, which were offered to the press institution for publication. Minor sequential adjustments of materials were made by the researchers according to the published paper textbooks. *The Integrated Coursebooks* are compiled with a content structure such that each book focuses on one practical issue as the “main theme.” Therefore, the language materials of each book are assorted into one sub-corpus. There are a total of four sub-corpora, and the total word count is 61,434.
2. Each sub-corpus was saved into plain text and uploaded onto the online corpus annotation tool Wmatrix 5.0 to be autonomously tagged.
3. Setting criteria for log-likelihood and cut-off, corpus results were swept for the target domain and source domain.
4. The semantic domains automatically annotated by Wmatrix for words in texts can roughly correspond to the source or target domains of conceptual metaphors (Sun, 2012, p. 8). Based on identifying which semantic domains serve as source or target domains, all lemmas and tokens belonging to those semantic domains can be extracted. The semantic annotation of the potential target domain is carried out using a broad sweep of “word” in Wmatrix to generate the word concordance in sentences, which is then followed by manual identification. Manual identification of linguistic metaphorical expressions by human raters is accompanied by two rounds of reliability testing. The kappa reliability rate is 0.6879.

## 5 Results and discussion

The following sections carry out the content analysis and seek deep insight into the above three research questions in the following logic: first, the metaphorical representations of knowledge contents in the textbooks are considered as “what the ‘SOURCE’ is”; second, the design feature (“PATH”) of metaphorical representation of knowledge contents in the textbooks is considered as the question of “how is it?”; third, the “GOAL” of the metaphorical representations is argued for the question of “why is it?”

### 5.1 SOURCE: metaphorical representations of knowledge contents in the texts and tasks

**Figure 1:**
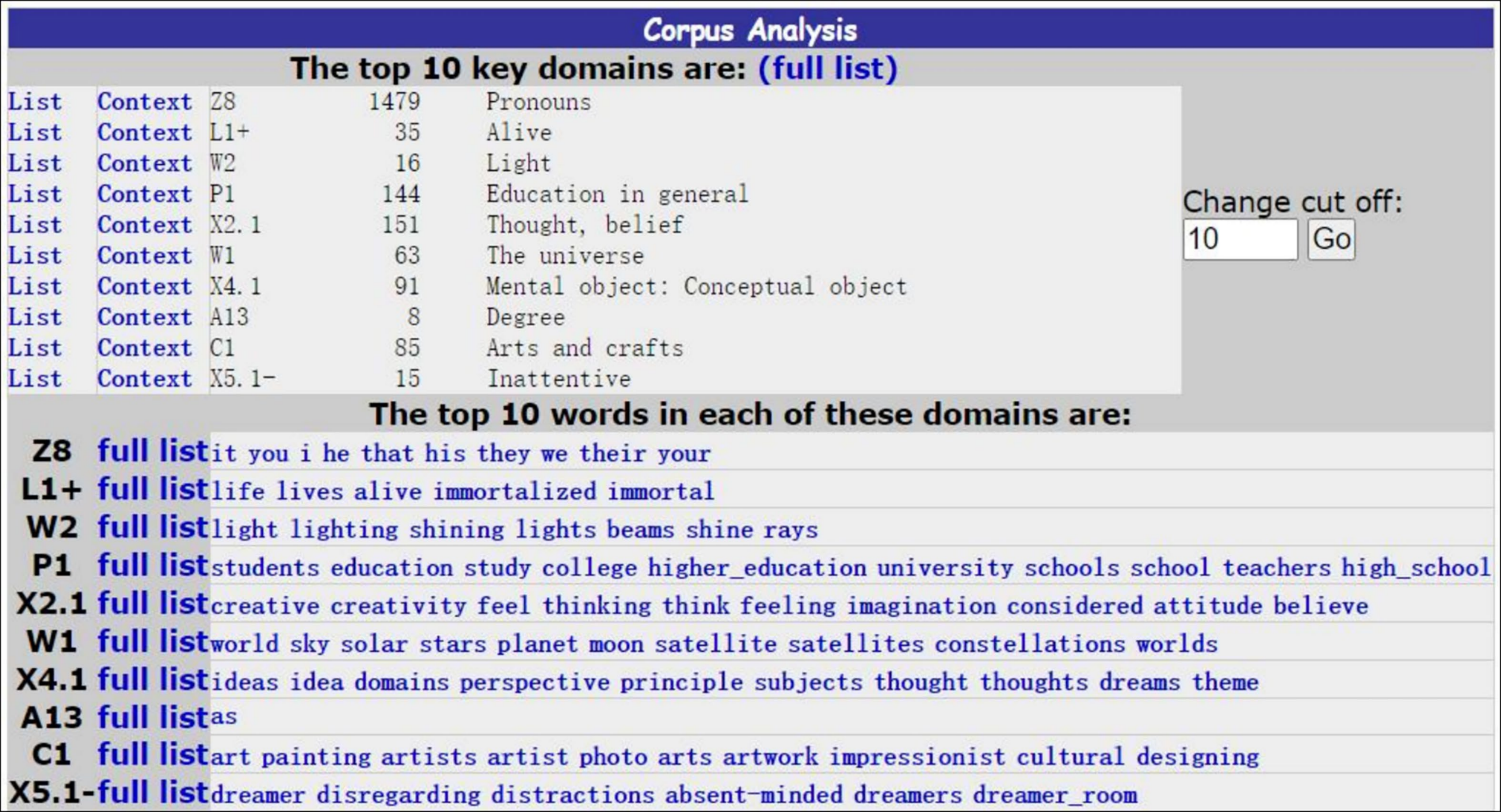
Semantic tagging for *the Integrated Coursebook One*.

Uploading one of the corpora in “.txt” format to online Wmatrix 5.0, the annotation is automatically completed by the CLAWS and USAS functions. Setting the keyness analysis compared to the BNC sampler written, results show the list of the possible source domains and target domains for each corpus in the Figure 1.

This platform offers a web-based interface for accessing a suite of natural language processing (NLP) tools, including the UCREL Semantic Annotation System (USAS) for English semantic analysis and the Constituent Likelihood Automatic Word-tagging System (CLAWS) for part-of-speech tagging. Among the top key domains, the one with the most abstract semantic annotation is picked as the target domain, and the one with the most concrete semantic annotation is treated as the source domain. Then the “context” showing the original sentence concordance is retrieved to locate the metaphor carrier, and then a “broad sweep” function is used to locate the word with reference to the original text. Findings in the text are shown in the following:

#### 5.1.1 Metaphorical representation of knowledge contents in the texts

In the corpus of Book One, excluding the grammatical functioning concept as “Z8” from the top list in Figure 1, the top concept domains in Book One fall on [P1]: education in general; [X2.1]: thought, belief; [X4.1]: mental object; [C1]: arts and crafts. Probing into the specific features in the word list for each concept domain, raters can determine the metaphorically loaned words concerning the conventional metaphor identification approach of MIPVU (Steen et al., 2010).

Findings show that the target domain is identical to the top concept domain in the corpus list as shown in Figure 1. Co-opting the Master Metaphor List (Lakoff et al., 1991), the top source domain falls on CONTAINERS, which is highly conventional according to CMT. A closer look into the context shows that the lexical carrier for the container metaphor is mostly verbs and prepositions. This result shows that conceptual metaphor reveals the embodiment in human cognition in a way that humans conceptualize the world through our experiences, for which embodied cognition takes new concepts in light of the objects in humans’ nearby space and forms a temporal scenario (as in a container). The other feature is that linguistic metaphors are embodied within linguistic chunks, as shown in the following example.

Example (1): *He says that whenever he felt that he had come to the end of the road or into a difficult situation in his work, he would take refuge in music.* (Students’ Book 1 Unit 3 Text A).

The above example was cited from the reading texts with linguistic metaphorical expressions to enumerate their features. The example presents a chunk that means seeking help or protection from someone when a difficult time comes. The human metaphor of “music” is activated to denote that he (Einstein as the protagonist) found his inner peace and tranquility in music; therefore, MUSIC IS CONTAINER gives a vivid presentation of the relationship between the protagonist and music.

Example (2): *Since then, he has set up at 40 festivals and will occasionally set up at DeVault Family Vineyards, where he’ll be this weekend for the watermelon festival.* (Students’ Book 2 Unit 2 Text B).

The second sentence employs the verb phrase “set up,” which is semantically categorized under [T2+: Time, beginning]. This stands in strong contrast to its typical usage with objects like “a tent” or “a ladder.” The observed meaning deviation suggests that this linguistic unit embodies a conceptual metaphor. The conceptual mapping of it is illustrated in the following:

(object) tent/building → fame.

(doer) agent → “he.”

(place)ground → festivals.

(action) physical set up → spiritually build up.

Manual identification reveals the underlying conceptual metaphors ‘A BUSINESS IS A BUILDING’ and ‘A FESTIVAL IS A BUILDING’, which are used here as fixed linguistic chunks. As established by scholars such as Lakoff and Johnson (1980/2003), metaphors are fundamental to how people conceptualize abstract ideas. Therefore, interpreting these conceptual metaphors within chunks offers learners advantages as follows: it serves as an efficient strategy for vocabulary acquisition and provides a cognitive framework for grasping thematic concepts. In this sentence, “set up” is used metaphorically to mean preparing for an event, not constructing a physical object. This semantic extension is corroborated by the Semantic Percentage Dictionary (Eurodic online: https://dict.eudic.net/), which shows the meaning has metaphorically extended from its primary sense of “create by putting components together” (accounting for 95% of usage) to the secondary sense of “get ready for a particular purpose or event” (accounting for 5%).

Confined to the genres of the selected texts, novel metaphors are fewer compared to conventional ones in *the Integrated Coursebooks*. Conventional metaphors are of pedagogical significance to CE learners in the following ways: First, language is deeply permeated by conventional metaphors in a way that they draw on familiar concepts and experiences, such as orientation and the human body, to make abstract concepts much easier to understand. Therefore, conventional metaphors embody the cognitive pattern through which humans interact with the world. Second, conventional metaphors are automatically processed by native speakers but might pose a challenge for CE learners (Boers and Lindstromberg, 2006), in the sense that they are culture-rooted and reflect shared beliefs, values, and experiences. The recognition and interpretation of these conventional metaphors would enable learners to become acquainted with the cultural context and thinking patterns of the target language, which would not only improve their intercultural communicative awareness but also enhance their critical thinking skills (Kastner-Hauler et al., 2022).

After the confirmation of metaphor carriers in the context sentences, the original reading articles were relocated, and the identification of source and target domains was conducted. These steps were carried out by the researchers. There are other examples of conceptual metaphors in the texts of the textbooks, summarized in the Table 2.

**Table 2:** Overall distribution of metaphors in the texts of *the Integrated Coursebook.*

Book	Total Freq.	Top source domain (Freq.)	Top target domain
Students’ Book 1	93	Container (15), object (11)	Education, ideas
Students’ Book 2	130	Container (26), possession (3)	Mental action, time
Students’ Book 3	131	Container (31), possession (10)	Relationship, body
Students’ Book 4	114	Human (6)	Science

Most of the metaphorical representations of knowledge contents in the texts take the form of conceptual metaphors, with conventional metaphors making up the majority. The tasks represent these with metaphorical meaning extension mechanisms. Conventional metaphors matter in language learning because they are used in a way that is closer to being native-like, as “metaphors being very well established by members of the discourse community, or even the most natural expressions, and used effortlessly by the communicator (Kövecses, 2010, p. 33–34),” although they map a less salient feature in the concrete domain (Banaruee et al., 2019). The ones in the texts are implicitly represented, while the ones in the tasks are explicitly designed. The examples are manifested in the next section.

#### 5.1.2 Metaphorical representation of knowledge contents in the tasks

The task design of *the Integrated Coursebooks* explicitly employs CMT as cognitive tools for vocabulary building.

Example (3): *In a metaphor, an analogy is made between two normally unrelated things or experiences to show how one resembles the other in one way or another. This is the way words normally get their meaning extended.*

The conceptual metaphor, as the underlying working mechanism of meaning extension, is designed in tasks of polysemous construction. The above task directives (He, 2007, p. 45) appear in the exercise of practicing the polysemy of “up” and “down.”

Another example is the translation task focusing on the “adjective + noun” construction. Chinese “*shen* (deep)” and English “fair” are selected contrastively to practice the multiple meanings in both source and target languages. One example is “*深井* (*shenjing*)” as translated into “a deep well”, while “*深交* (*shenjiao*)*”* is equivalent to “long and intimate friendship”. “Fair” in “a fair deal” is *“合理* (*heli*)*”* in Chinese, while “fair” in “fair hair” is translated into “*深色* (*qianse*)”. The metaphorical meaning extension mechanism in the tasks could provide linguistic motivation for meaning extension of the same word, and construction might help to enhance learning efficiency.

Metaphorical representations of knowledge contents in the tasks also make use of analogical reasoning to design the writing tasks with sample writings. Such practice is common in college English textbooks published in China. Analogy, as a specific manifestation of metaphor in rhetoric, is widely recognized as a crucial cognitive tool and reasoning process that facilitates the production and comprehension of conceptual metaphors, and it is used as a teaching facilitator to aid the teaching of abstract subjects (Jonsson et al., 2020). Gentner (1983, p. 162) contended that metaphors grounded in relational similarity are, in essence, analogical in nature. In Gentner and Gentner (1983, p. 100), the authors proposed a theoretical framework for analogical thinking, identifying structural mapping as a cognitive mechanism that operates similarly to the conceptual metaphor theory advanced by Lakoff and Johnson (1980/2003).

**Figure 2:**
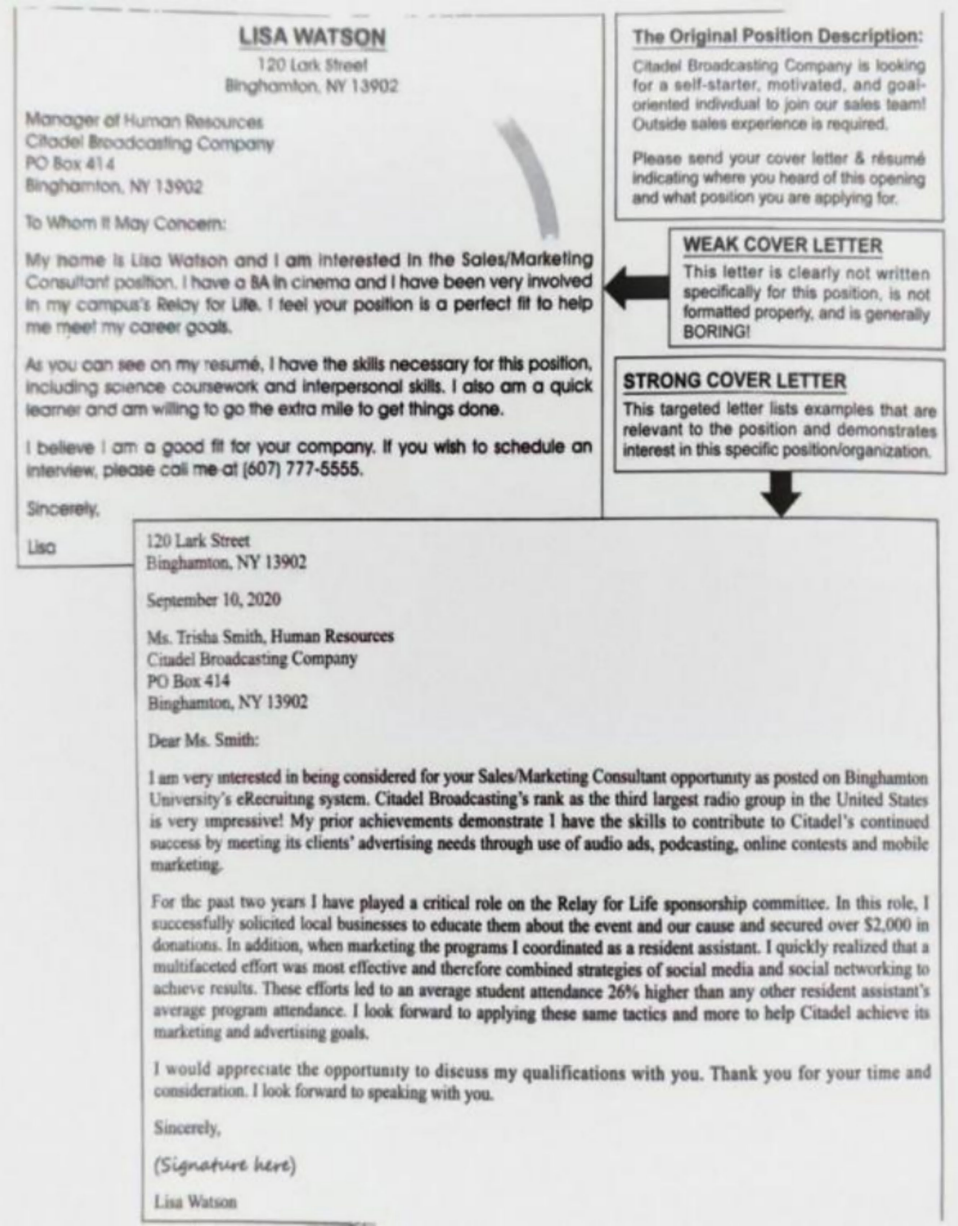
Illustration of cover letter in writing task Students’ Book P.64.

Xu (2018, p. 16), meanwhile, emphasized that “analogical metaphor represents a distinctive metaphorical feature of the Chinese language.” He elaborated on the universality of metaphor alongside the specificity of analogy, highlighting its role in enabling cross-domain mappings between disparate categories. Such mappings, as Xu argued, establish the foundation for event proposition formation and are realized through analogical reasoning—a cognitive process driven by the interplay of imagination and association (Figure 2).

The following is an example from *the Integrated Coursebooks*.

Example (4): *The following illustration (Figure 2) shows us a sample cover letter for a job application.*

Equipped with intuitive knowledge, imitation enables learners to use analogical reasoning to write application letters. College English textbooks could incorporate more allusions, anecdotes, stories, and examples to enhance analogical reasoning in language learning, particularly for skill acquisition. For instance, *The Integrated Coursebooks* design writing tasks in Unit Seven of Book Two to develop argumentative paragraphs.

### 5.2 PATH: metaphorical representations of knowledge contents in the unit with structured design

**Figure 3:**
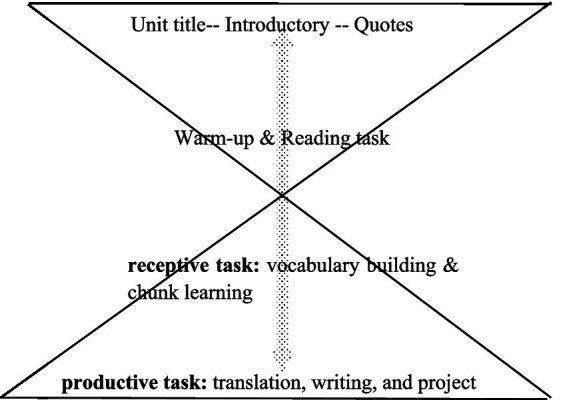
The hourglass structural representation of the unit.

The thematic realization in *the Integrated Coursebooks* employs metaphorical analogies to frame abstract concepts as embodied experiences, enabling students to “learn through experiencing” (Figure 3).

Overall, the metaphorical representation of knowledge contents in the unit with structured design shows an “hourglass” pattern, which is first claimed as a “general-specific-comprehensive” design pattern by the chief compiler of the textbooks (Liu and Shi, 2022, p. 201). This design pattern is positively manifested in this study, and is metaphorically graphed as follows:

The four books of *the New Target College English Integrated Coursebooks* have the same unit structure and layout, which also display the same structural design pattern as an “hourglass.” Table 3 are two units in *Book Two* that exhibit the patterned structure as a universal feature of the textbooks:

In Table 3, *Unit Two of Book Two* mainly focuses on the topic of “business,” the language skill of “comparing,” and the language expression of polysemous construction. These elements are integrated into the unit theme, and through a repeated design of tasks on the three aspects, the unit forms an hourglass structural pattern. From the upper bulb of the hourglass, the massive topic of “business” is narrowed down to Text A on “traits” and Text B about “a story.” Through the neck, linguistic knowledge is filled out and practiced. At the bottom, writing and projects employ output tasks of writing and team presentations to practice and reinforce the three aspects of knowledge as well as skills in a comprehensive manner. *Unit Five* in the table displays the same pattern. Detailed elaboration is in the following paragraphs:

**Table 3:** Universal design as “Hourglass” structural representation in *Book Two.*

Structure of hourglass	Structure of the unit	Unit 2	Unit 5
Hourglass upper bulb	Unit title	Gaining a foothold in the business world	Studying abroad
	Introductory	Information about running a business start-up	Brief introduction to studying in the UK and US.
	Quotes	Steve Jobs & Confucius’ words about opportunity and teamwork	Henry Miller And A Chinese educator’s words about study and travel
Hourglass Neck	Warm-up	Discussion on “entrepreneur”	Michelle Obama’s speech on some advantages of studying abroad
	Reading task	Text A about traits of “entrepreneur”; Text B about the story of “A 13-Year-Old Entrepreneur”	Text A on student’s life in Paris; Text B about American student at Cambridge
Hourglass Lower bulb	Reading comprehension	Blank-filling to compare “entrepreneurs and managers”; T/F and open questions on text A’s comprehension;	Blank-filling on specific time events and experience in Paris
	Vocabulary & translation	word-filling and grammar from paragraphs in text A; “run+noun” polysemous construction; a polysemous word as antonyms; translation of the expressions of comparison and contrast	Blank-filling in passage talking about benefits of studying abroad; word-filling and grammar from paragraphs in text A; “noun+ to do” construction; metaphorical meaning of “up & down”, “hot & fire”; translating “there+ be” construction
Hourglass bottom	Writing	About comparison/contrast paragraphs	Paragraphs in chronological order and the use of a flashback
	Project	Teamwork about organizing students’ club such as entrepreneurship club	Group-work on a guideline for studying abroad

Taking another example, Text A of Book 3, Unit 6 is titled *“Life as You See* It.” It addresses personal attitudes toward life—a topic highly relevant to college students navigating a rapidly changing world, yet abstract for those with limited real-world experience. The unit’s theme introduction constructs meaning with conceptual metaphors such as THE WORLD IS YOURSELF, NEGATIVE EMOTION IS A PLACE ONE FREQUENTS, and DEPRESSION IS POSSESSION. These metaphors collectively conceptualize the theme by suggesting that happiness or depression depends on the “place” one chooses to inhabit mentally, which is a common metaphor as emotions are often described in this way (Khatin-Zadeh et al., 2017). Knowing how to deal with negative emotions totally depends on your reaction, and it can absolutely be treated positively, thereby encouraging positive thinking and strong emotional resilience.

The thematic introduction is followed by two complementary quotations—one from the Chinese classic *I Ching*, and the other by William James—both reinforcing the value of tenacity and self-motivation in cultivating a positive outlook on life. The follow-up text materials offer a visual presentation of these components.

The unit begins by introducing depression in a warm-up section, followed by oral practice exploring potential causes of depression among college students. To address learners’ possible apprehensions, a narrative essay structured in a “problem–solution” format is included as the reading task. This essay recounts the author’s personal experience of overcoming depression, allowing readers to empathize with the author’s spiritual journey and develop their own problem–solution awareness. With deep engagement, key concepts are internalized and reinforced via mental imagery and repeated practice, forming an interconnected knowledge network.

After reading, tasks are followed to extend from the reading materials and to integrate language skill development, critical thinking, and intercultural awareness. Receptive tasks (Nation, 1990) focus on chunk learning of “verb + noun” constructions, while productive tasks reinforce linguistic structures and promote intercultural competence through translation between source and target languages. This integrated approach supports the incremental acquisition of vocabulary—from receptive to productive mastery (Lyu et al., 2022). Throughout the unit, human metaphors are used to conceptualize depression as a person who is mysterious and unfamiliar, making abstract notions more relatable.

Metaphorical representation with structured design employs embodied cognition effectively in a way that can enhance learning efficiency through self-experience. Research has shown that human language acquisition “use an analogy to relate similar entities to the first relevant iconic object they encounter through a process of subsumption simulated by perceptual Gestalt (Banaruee et al., 2023, p. 3).” Human metaphors coherently enable the experiential perception (Chen and Yu, 2022), while tasks designed with text materials practiced and reinforced the trinity of knowledge contents.

This compilation makes use of a systematic structured design to coherently integrate the reading materials with task drilling. The human metaphor is repeatedly employed to construct the meaning that, though depression is initially strong to you, upon knowing, he/she might eventually become an acquaintance. The conceptualization of the unit theme is represented in this way and in a holistic manner throughout reading to tasking.

### 5.3 GOAL: metaphorical realization of teaching objectives

Aligned with the National Teaching Guidelines and the advocacy for morality education, *the New Target College English Integrated Coursebooks* establish “three-in-one” teaching objectives to develop learners’ language competence, critical thinking, and intercultural competence.

Among the three objectives, language competence serves as the primary and foundational pillar. The development of trinity competence is predicated on the understanding that a solid command of language structures is a prerequisite for fostering critical thinking—encompassing both the disposition and skills as defined in the “Delphi Report” (Facione, 1990). As Liu and Shi (2022, p.163) proposed, such thinking ultimately emerges from solid language mastery. Concurrently, moral cultivation is seamlessly interwoven throughout the language learning process, whether by shaping a constructive attitude toward life’s challenges or by guiding students in the appreciation of artistic works.

In the fulfillment of the teaching objectives, metaphorical representations serve as the lubricant and, meanwhile, a pathway to realize the design in textbook development. It functions in the following way:

First of all, language competence could be efficiently trained through chunk learning and linguistic construction in the textbooks, and conceptual metaphors provide cognitive motivation to help learners achieve cognitive equivalence and fluency. The centrality of meaning induces a dynamic view of grammatical structures. Any change in form will lead to a meaning shift; however, it is fundamentally motivated and interconnected through metaphorical mechanisms. Since language is usage-based, the rigid application of grammar rules may not be accurate, as language is subjective and varies according to contexts or speech intentions. The development of metaphorical competence could enhance conceptual fluency (Danesi, 1995) and help achieve cognitive equivalence.

Second, since language is the expression of thought, learning a language and mastering linguistic structures could ultimately lead to the training of thinking ability. The mastery of the laws of language contributes to criticality in an unnoticeable way. In the process, conceptual metaphor helps to establish the network of meaning and linguistic structures and offers motivation for its relational change, which is effective in developing learners’ dialectical, abstract, and systematic thinking abilities. The language knowledge network also induces creativity and imagination. For example, in Unit “Life as You See It”, the conceptual metaphor DEPRESSION IS POSSESSION gives learners a different perspective on the issue of depression, viewing it as a common object that everyone may possess, with the only matter being how people choose to act on it. A different but dialectical perspective on “the issue of depression” endows learners with a rational and divergent thinking pattern that, through practice, would be beneficial for the training of critical thinking.

Third, intercultural competence could be trained with upright cultural views and intercultural awareness. Through observing, appreciating, and comparing the target language and the source language, cultural commonalities and differences are recognized. With cultural comparison and experience of diversified cultural conduct, proper attitudes toward common issues, a broader horizon of intercultural occasions, and sound judgment on trending topics will be established. In the process, the cultural connotation that conceptual metaphor carries in language content can boost a deeper understanding of cultures and trigger diverse perspectives on international issues, problems, or conflicts.

As illustrated in Unit One of Book One, the conceptual metaphor LIFE IS A JOURNEY is employed to narrate the exile experiences of the Chinese composer *Xian Xinghai*. During his time abroad, Xian encountered various hardships and immersed himself in the lives of people from other cultures, ultimately composing several monumental musical works. In contrast, another cultural figure in this unit, *Albert Einstein*, also an avid music enthusiast, regarded music as a devoted friend from whom he sought emotional solace. Despite their different approaches—Xian drawing inspiration from life’s struggles while Einstein turned to music for inner comfort—both characters placed profound value on music.

As a universal art form, music transcends cultural boundaries. When people from diverse backgrounds share the same melodies and resonate with similar emotions, their intercultural awareness is naturally enhanced. Through the lens of these two distinct lives, we see how music serves not only as personal sustenance but also as a bridge to mutual understanding.

All in all, the training of language competence, critical thinking, and intercultural competence is inextricably intertwined and can be realized essentially with language competencies in textbook development through a metaphorical pathway. Textbook compilation and design utilizing a metaphorical representation could potentially achieve the teaching objectives in an effective and well-organized way.

## 6 Conclusion and implications

Based on the study’s findings and discussion, explicit teaching of metaphor through comparison is advocated here. Low (1988, p. 137–138) suggested that conventional metaphors are practiced in a “polysemy method,” in which multi-text comprehension of one single source domain develops metaphorical awareness of structuring and boundaries of the metaphor, such as “root.” Meanwhile, the teaching of innovative metaphor is prone to production extended on a comprehensive comprehension of the conventional use of the same concept. Exemplars are given with “the systematicity of metaphor”, which is also exemplified in the *Integrated Coursebooks*. Liu and Shi (2022, p. 133-139) emphasized the importance of teaching metaphorical expressions for the development of intercultural competence. It is also claimed that comparison is the most effective way of teaching metaphor. *The Integrated Coursebook* designed tasks on linguistic structures in comparison between Chinese polysemous structures and English ones. It is also considered an implementation of content juxtaposition.

In conclusion, this study analyzes *the Integrated Coursebooks* based on a “SPG” model in light of applied cognitive linguistics and conceptual metaphor theory, and tries to probe into the function of metaphors in textbook development.

Findings show that first, conceptual metaphors pervade language materials in the textbook, and the metaphorical representations of knowledge contents in the text and tasks take the form of conceptual metaphors, metaphorical extension mechanisms, and metaphorical analogical reasoning. Language materials predominantly feature conventional metaphors, and the representation of knowledge contents occurs in both implicit and explicit manners.

Second, metaphorical representations of knowledge contents are designed with structured thinking in the textbook to form an “hourglass” pattern of unit organization. The complex process of knowledge structuring relies on the dynamic interplay between structured thinking and metaphorical representation. Structured thinking initiates this process by providing a disciplined cognitive framework, which in turn enables the construction of coherent metaphorical models. These models are pivotal for integrating disparate pieces of information; they create meaningful connections through sophisticated cognitive operations such as cross-domain mapping, analogical reasoning, and the establishment of conceptual interconnections. By translating abstract or fragmented concepts into more intuitive, often embodied formats, metaphorical representations make them amenable to mental manipulation. Ultimately, this synergy between structured thinking and metaphor facilitates the emergence of a hierarchical knowledge architecture. This architecture is not arbitrary but is deeply rooted in the principles of embodied cognition and is constrained by the inherent mechanisms of the human cognitive system, ensuring that the resulting knowledge structures are coherent, robust, and adaptable.

Third, the function of such metaphorical representations is to realize the textbooks’ teaching objectives, as in the *Integrated Coursebooks* the development of language, critical thinking, and intercultural competences. The three aspects are mutually boosted and neatly intertwined. They are cultivated in a holistic way and can be realized through a metaphorical pathway: The “three-as-one” objectives in the textbooks are easily realized through metaphor teaching and learning for several reasons. First, conceptual metaphor is a cognitive way of being human and is ubiquitous in linguistic expressions; the teaching emphasizing understanding and production of conceptual metaphor is both convenient and accessible. Second, metaphorical awareness and competence is a higher-order thinking competence and is a relatively overt indicator of critical thinking. Third, conceptual metaphors are mostly socio-cultural in that they can reflect the thinking patterns of a social group; thus, a good mastery of them might boost intercultural awareness and intercultural competence.

To further gain a better understanding of the instructional effectiveness of metaphorical representations in textbooks and the development of metaphorical competences, classroom experiments are to be carried out to collect empirical evidence for this research. Cross-country or cross-textbook comparisons would be carried out to examine how robust the SPG-based findings in this study are.

## Data Availability

The original contributions presented in the study are included in the article/supplementary material, further inquiries can be directed to the corresponding author.
